# Squamous Cell Carcinoma Arising From Mature Cystic Teratoma in a 39-Year-Old Woman: A Case Report

**DOI:** 10.7759/cureus.110609

**Published:** 2026-06-10

**Authors:** Thaleia Alexandra Ananika, Maria Roxani Koutrouli, Iakovos Vlachos, Nikolaos Dafnios, Despoina Myoteri, Vasiliki Michalaki

**Affiliations:** 1 Second Department of Surgery, Aretaieion University Hospital, National and Kapodistrian University of Athens School of Medicine, Athens, GRC; 2 Department of Pathology, Aretaieion University Hospital, National and Kapodistrian University of Athens School of Medicine, Athens, GRC; 3 Department of Oncology, Aretaieion University Hospital, National and Kapodistrian University of Athens School of Medicine, Athens, GRC

**Keywords:** dermoid cyst, mature cystic teratoma, ovarian carcinomas, ovarian squamous cell carcinoma, surgical case report

## Abstract

Ovarian carcinomas represent the second most common malignancy of the female genital tract; however, primary squamous cell carcinoma (SCC) is a rare subtype. Most of these tumors arise from malignant transformation of mature cystic teratoma (MCT), whereas others are associated with endometriosis or Brenner tumor. Preoperative diagnosis remains challenging because both clinical presentation and imaging findings are frequently nonspecific. Patients often present with symptoms related to the mass effect of the lesion, and metastatic disease may be present at the time of diagnosis. While pelvic ultrasonography and magnetic resonance imaging (MRI) are essential diagnostic adjuncts, histopathological examination remains the gold standard for establishing the diagnosis and guiding management. Current therapeutic strategies necessitate a multimodal approach, including surgical debulking and adjuvant chemotherapy or radiotherapy. We present the case of a 39-year-old woman diagnosed with ovarian SCC arising from a mature cystic teratoma, detailing her clinical presentation and subsequent management. This case highlights the diagnostic and therapeutic challenges associated with malignant transformation of MCTs, particularly in younger patients.

## Introduction

Mature cystic teratomas (MCTs), also known as dermoid cysts, are the most common ovarian germ cell tumors in women of reproductive age. Although the majority are benign, malignant transformation may occur in approximately 1%-2% of cases, with squamous cell carcinoma (SCC) representing the most common histological subtype. Ovarian SCC arising from MCT is rare, accounting for less than 1% of ovarian malignancies [1]. Malignant transformation usually occurs in postmenopausal women between 45 and 60 years of age and is frequently associated with large tumor size [2]. Clinical manifestations are often nonspecific and may include abdominal pain, abdominal distension, urinary or gastrointestinal symptoms, and the presence of a pelvic mass. Imaging findings may suggest malignant transformation; however, definitive diagnosis relies on histopathological examination [1].

The rarity of this entity has limited the development of standardized diagnostic and therapeutic protocols. Hence, most cases are diagnosed postoperatively [3]. Most treatment strategies are extrapolated from epithelial ovarian cancer management and generally involve extensive surgical resection combined with platinum-based chemotherapy. Prognosis depends largely on disease stage and completeness of surgical cytoreduction. However, because the tumor often presents at an advanced stage due to the lack of early symptoms, the overall prognosis can be poor [4].

We present the case of a 39-year-old woman diagnosed with SCC arising from a mature cystic teratoma, emphasizing the diagnostic difficulties, surgical management, pathological findings, and postoperative follow-up.

## Case presentation

A 39-year-old woman presented to our department with atypical abdominal pain, abdominal discomfort, vomiting, progressive abdominal distension, urinary frequency, and menstrual cycle disturbances. She was gravida 0, para 0, with no significant past medical history. Initial pelvic ultrasonography demonstrated a large pelvic lesion measuring approximately 13 × 14 cm and appearing to arise from the uterus. Magnetic resonance imaging (MRI) revealed a large heterogeneous pelvic mass measuring approximately 14 × 15.5 × 16 cm, causing compression of the ureters (Figure 1). In direct contact with the superior aspect of this lesion, a second mass was identified. This second mass measured up to 16 cm in its greatest dimension and demonstrated both solid and cystic components, with areas containing fatty tissue (Figure 2). 

**Figure 1:**
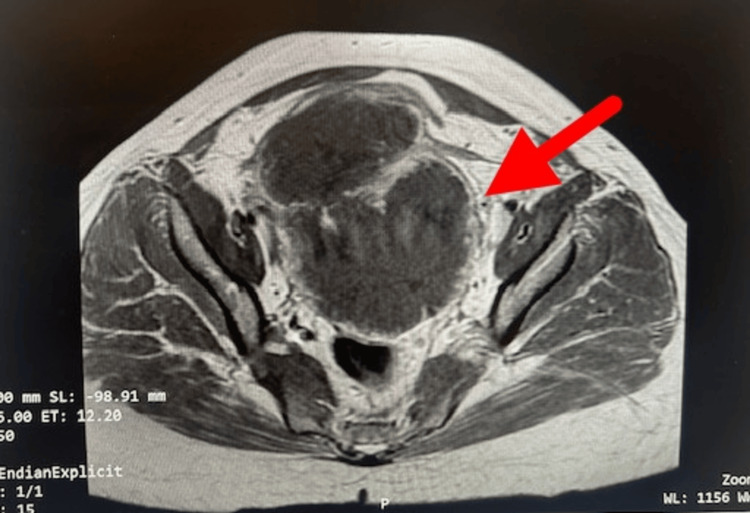
MRI demonstrating a large heterogeneous pelvic lesion within the lesser pelvis causing compression of adjacent structures (red arrow)

**Figure 2:**
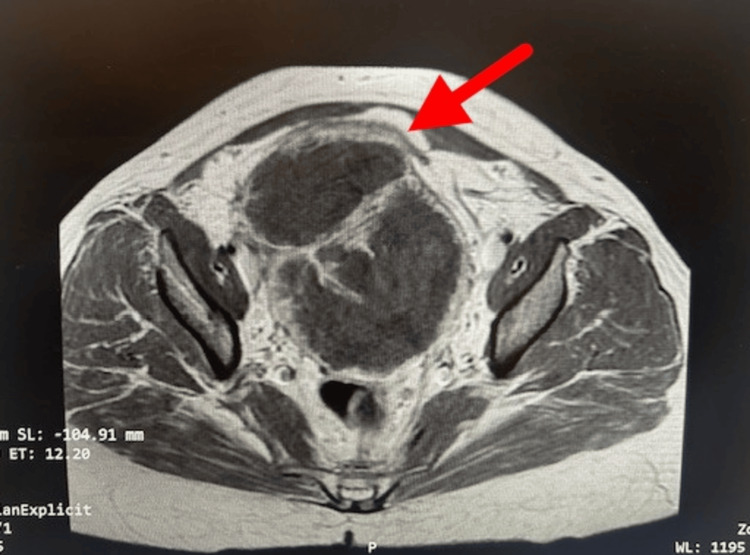
MRI demonstrating heterogeneous tissue components with fatty areas suggestive of mature cystic teratoma (red arrow)

MRI also demonstrated ascites and findings suspicious for peritoneal implants within the pelvis. The radiological appearance initially created uncertainty regarding whether the findings represented a single locally advanced pelvic lesion or multiple contiguous masses. Given the extensive pelvic involvement and ureteric obstruction, malignancy was strongly suspected.

Following imaging studies, the patient underwent paracentesis of the mass, and the specimen was submitted for histopathological examination, which revealed squamous cell carcinoma. Because of worsening obstructive uropathy and concern regarding renal impairment, a right percutaneous nephrostomy with stent placement was initially performed. Two days later, the nephrostomy was removed, and bilateral ureteral pigtail stents were inserted.

Laboratory evaluation revealed a serum creatinine level of 1.4 mg/dL, albumin 3.2 g/dL, sodium 135 mmol/L, total calcium 14.6 mg/dL, C-reactive protein 16.2 mg/L, and LDH 208 U/L. Complete blood count demonstrated microcytic hypochromic anemia with hemoglobin 9.3 g/dL, mean corpuscular volume 73.9 fL, and mean corpuscular hemoglobin 22 pg. Thrombocytosis was also present, with a platelet count of 452 × 10³/μL. Tumor marker evaluation demonstrated elevated CA-125 (124.3 U/mL), CA 19-9 (83.8 U/mL), and HE4 (391.4 pmol/L), whereas carcinoembryonic antigen and alpha-fetoprotein levels were within normal limits (Table 1). 

**Table 1:** Results of the laboratory evaluation

Parameter	Value	Reference Range
Serum creatinine	1.4 mg/dL	~0.6-1.2 mg/dL
Albumin	3.2 g/dL	3.5-5.0 g/dL
Sodium	135 mmol/L	135-145 mmol/L
Total calcium	14.6 mg/dL	8.5-10.5 mg/dL
C-reactive protein (CRP)	16.2 mg/L	<5 mg/L
Hemoglobin	9.3 g/dL	12-16 g/dL (female)
Mean corpuscular volume (MCV)	73.9 fL	80-100 fL
Mean corpuscular hemoglobin (MCH)	22 pg	27-33 pg
Platelets	452 ×10³/μL	150-400 ×10³/μL
Cancer antigen-125 (CA-125)	124.3 U/mL	0-35 U/mL
Cancer antigen 19-9 (CA 19-9)	83.8 U/mL	0-37 U/ml
Carcinoembryonic antigen	Within normal limits	
a-Fetoprotein	Within normal limits	
Human epididymis protein-4 (HE-4)	391.4 pmol/L	Reference range varies, but it is considered elevated
Lactate dehydrogenase (LDH)	208 U/L	135-214 U/L

Subsequently, the patient underwent exploratory laparotomy. Intraoperatively, a large pelvic tumor occupying the adnexal region and densely adherent to the uterus and rectosigmoid colon was identified. Total hysterectomy with bilateral salpingo-oophorectomy, iliac lymph node dissection, infracolic omentectomy, and partial rectal resection with end colostomy were performed because of suspected local invasion and extensive adhesions.

Gross pathological examination demonstrated a 23 × 20 × 12 cm partially solid and partially cystic mass containing hair and sebaceous debris, compatible with mature cystic teratoma. Histopathological examination confirmed well- to moderately differentiated squamous cell carcinoma arising from mature cystic teratoma (Figure 3). Microscopic examination demonstrated keratinizing squamous epithelium with transition to high-grade dysplasia and invasive SCC infiltrating the ovarian parenchyma (Figures 3, 4). Sebaceous glands, adipose tissue, smooth muscle, and neural tissue were also identified within the teratomatous components (Figure 3). Examination of the lymph nodes, omentum, cervix, and rectal specimen demonstrated no evidence of metastatic disease. The tumor was staged as FIGO stage IIB (T2b). Postoperatively, the patient received six cycles of adjuvant chemotherapy with paclitaxel (175 mg/m²) and carboplatin (AUC 5), administered every 21 days.

**Figure 3:**
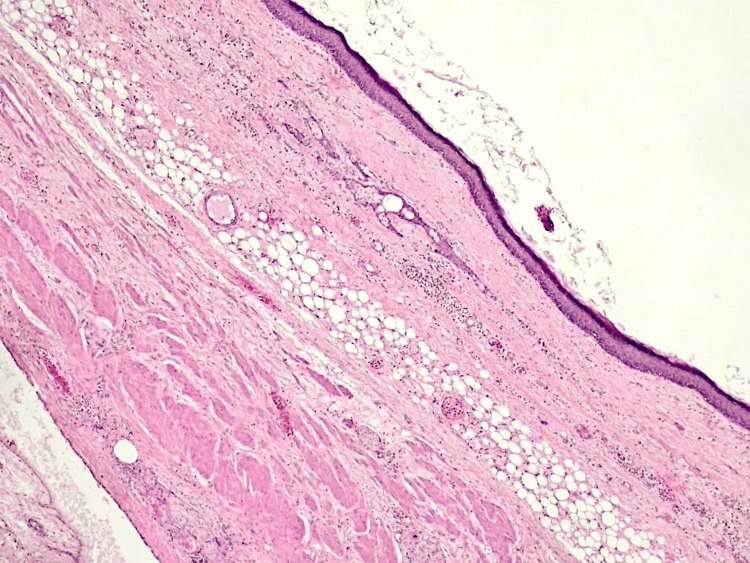
Section of the mature cystic teratoma wall, showing an inner layer of keratinizing squamous epithelium. Sebaceous glands, adipose tissue, smooth muscle, and nerves are also visible (H-E stain, ×40 magnification)

**Figure 4:**
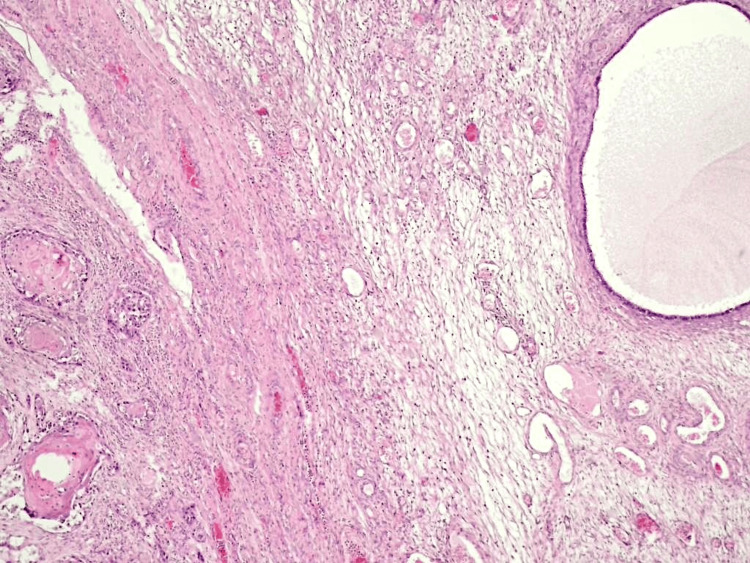
Keratinizing squamous cell carcinoma (left side of image) infiltrating the parenchyma of the ovary (ovarian inclusion cyst visible at the right side of the image) (H-E stain, ×40 magnification)

Twelve months after surgery, PET imaging demonstrated increased FDG uptake in a left medial iliac lymph node (SUVmax 7) and para-aortic lymph nodes (SUVmax 2), raising concern for recurrent disease. Subsequently, restoration of gastrointestinal continuity was performed. During the same procedure, suspicious lymph nodes and a lesion involving the left ureter were excised and submitted for frozen-section and formal histopathological examination.

Histopathological examination demonstrated inflammatory changes without evidence of recurrent malignancy. Biochemical parameters and tumor markers had normalized during follow-up. The patient remains disease-free and is currently enrolled in a structured follow-up protocol, including MRI every six months. 

## Discussion

Mature cystic teratomas (MCTs), also known as dermoid cysts, are the most common ovarian germ cell tumors in women of reproductive age [1]. They account for approximately 10%-20% of all ovarian tumors and are bilateral in 10%-17% of cases [2,5], with an estimated incidence ranging from 1.2 to 14.2 per 100,000 women per year [6].

MCTs originate from primordial germ cells and therefore may contain differentiated tissues derived from all three germ layers [1,2,6]. These components can include ectodermal elements such as epidermis and sebaceous or sweat glands; mesodermal elements such as teeth, muscle, fat, and bone; and endodermal tissues, including gastrointestinal, respiratory, and thyroid epithelium [1,7]. Most mature cystic teratomas are benign; however, malignant transformation represents a rare but clinically significant adverse event, typically occurring in women between 45 and 60 years of age [8,9]. Variations in the reported age distribution have been described in the literature. Chen et al. reported a mean age at diagnosis of 55 ± 14.4 years, with 66.8% of patients being at least 50 years old at the time of diagnosis [10]. Similarly, Hackethal et al. reported a mean diagnostic age of 55 years [11]. The reported incidence of malignant transformation ranges from 0.5%-3% [1] to 1%-2% [2,6,7,12]. Malignant transformation may arise from any of the germ cell-derived components present within the tumor; however, squamous cell carcinoma is the most common histological subtype, accounting for up to 80% of cases [1,5-7], with some studies reporting even higher percentages (88.9%) [2]. Other reported histological types include adenocarcinomas, melanomas, carcinoid tumors, oligodendrogliomas, and sebaceous carcinomas, although these entities are exceedingly rare [1,7,12]. The present case is notable because the patient was only 39 years old, considerably younger than the age group most frequently described in the literature.

Preoperative diagnosis remains difficult because signs and symptoms are generally nonspecific and frequently reflect mass effect on adjacent pelvic organs. These signs and symptoms may include abdominal pain and the presence of a palpable abdominal or pelvic mass [9], although many patients remain asymptomatic or experience minimal symptoms. Abdominal pain has been reported in 50%-58.1% of cases, while a mass is identified in 31%-71.6% of patients [11]. Other symptoms related to invasion of or pressure on adjacent organs include vaginal bleeding, constipation or diarrhea, and urinary frequency or retention [5,8]. Systemic manifestations such as fever, weight loss, and cachexia have also been reported [6]. In our patient, progressive abdominal distention, urinary frequency, vomiting, and ureteric obstruction were among the dominant clinical manifestations. Bilateral ureteric compression resulted in obstructive uropathy requiring urgent urinary decompression before definitive surgery.

Regarding the diagnostic approach, it is considered that every ovarian tumor should be considered malignant until proven otherwise [11]. MRI and ultrasonography are considered the standard imaging procedures [1,2,13]. Tumor size and specific imaging characteristics on ultrasonography and MRI may aid in distinguishing malignant squamous cell carcinoma transformation arising in a mature cystic teratoma. MCTs associated with SCC are typically larger than benign MCTs [14].

According to Kikkawa et al., tumors with a diameter greater than 9.9 cm or those demonstrating rapid growth may be associated with an increased risk of malignant transformation [15]. Moreover, Chen et al. reported that 78.7% of MCTs with SCC were larger than 10 cm, with a mean tumor size of 13.8 cm [10]. Radiological signs of intratumoral vascularization are also indicative of malignant transformation of the teratoma [12].

However, histopathological examination remains essential for definitive diagnosis [8]. In our patient, the coexistence of teratomatous elements and invasive keratinizing SCC confirmed malignant transformation within an MCT. Importantly, no metastatic involvement was identified in the lymph nodes, omentum, cervix, or rectal specimen despite the extensive local disease.

Tumor markers may contribute to clinical suspicion but lack adequate specificity [14]. Certain tumor markers may be elevated, indicating the presence of malignancy. These tumor markers include SCC antigen, CA-125, CA 19-9, and CEA [2,11,13,16]. It has also been suggested that high levels of CA-125 are a more reliable prognostic marker than SCC antigen. Chen et al. reported that patients with elevated SCC antigen and CA-125 levels had a worse five-year survival rate than those with normal values [10]. In the present case, elevated CA-125, CA 19-9, and HE4 levels supported the suspicion of ovarian malignancy.

Surgical management remains the cornerstone of treatment. The standard primary treatment consists of total hysterectomy with bilateral salpingo-oophorectomy [1,5,14]. An open surgical approach is generally preferred in order to minimize the risk of tumor spillage [7]. In cases of early-stage disease, comprehensive surgical staging is recommended and includes omentectomy, peritoneal biopsies, and pelvic and/or para-aortic lymph node dissection. In advanced-stage disease, cytoreductive surgery is performed. In selected young patients who wish to preserve fertility, unilateral salpingo-oophorectomy combined with appropriate surgical staging may be considered. In our patient, dense adhesions and suspected rectosigmoid invasion necessitated partial rectal resection and end colostomy in addition to total hysterectomy, bilateral salpingo-oophorectomy, iliac lymph node dissection, and infracolic omentectomy.

Because no standardized treatment guidelines exist, adjuvant platinum-based chemotherapy is commonly administered. The combination of paclitaxel and carboplatin is the most commonly used regimen and was selected for our patient. PEB (cisplatin, etoposide, and bleomycin), which is commonly administered for germ cell tumors, has shown limited efficacy in the management of the squamous cell carcinoma component [12]. The role of radiotherapy or combined chemoradiotherapy remains controversial [6,7].

In our patient, follow-up PET imaging demonstrated FDG-avid lymph nodes, initially raising concern for recurrence. However, subsequent surgical excision and histopathological analysis demonstrated inflammatory rather than malignant changes. This finding highlights the limitations of imaging during postoperative surveillance and emphasizes the importance of histological confirmation before establishing recurrent disease.

Squamous cell carcinoma arising from a mature cystic teratoma is associated with a poorer prognosis compared with epithelial ovarian cancer [7]. Prognosis is primarily influenced by disease stage, particularly stage I, which represents a favorable prognostic factor [11], as well as by the completeness of cytoreduction [12]. According to Chen et al., the overall five-year survival rate across all stages is 48.4%. Stage I disease demonstrates the most favorable outcome, with a five-year survival rate of 75.7%, followed by stage II at 33.8%, stage III at 20.6%, and stage IV at 0% [10]. Therefore, long-term surveillance remains essential, and patients should be enrolled in a follow-up protocol [17].

## Conclusions

Primary ovarian squamous cell carcinoma arising from a mature cystic teratoma is a rare and diagnostically challenging malignancy. Large heterogeneous pelvic masses, obstructive uropathy, elevated tumor markers, and hypercalcemia should raise suspicion for malignant transformation. Histopathological examination remains essential for definitive diagnosis. Management requires a multidisciplinary approach involving extensive surgery and adjuvant chemotherapy. This case additionally highlights the importance of careful postoperative surveillance and histological confirmation of suspected recurrence.
